# Network Intrusion Detection Based on a General Regression Neural Network Optimized by an Improved Artificial Immune Algorithm

**DOI:** 10.1371/journal.pone.0120976

**Published:** 2015-03-25

**Authors:** Jianfa Wu, Dahao Peng, Zhuping Li, Li Zhao, Huanzhang Ling

**Affiliations:** 1 College of Automation, Harbin Engineering University, Harbin, Heilongjiang, China; 2 College of Computer Science and Technology, Harbin Engineering University, Harbin, Heilongjiang, China; 3 College of National Secrecy, Harbin Engineering University, Harbin, Heilongjiang, China; 4 College of Science, Harbin Engineering University, Harbin, Heilongjiang, China; Semmelweis University, HUNGARY

## Abstract

To effectively and accurately detect and classify network intrusion data, this paper introduces a general regression neural network (GRNN) based on the artificial immune algorithm with elitist strategies (AIAE). The elitist archive and elitist crossover were combined with the artificial immune algorithm (AIA) to produce the AIAE-GRNN algorithm, with the aim of improving its adaptivity and accuracy. In this paper, the mean square errors (MSEs) were considered the affinity function. The AIAE was used to optimize the smooth factors of the GRNN; then, the optimal smooth factor was solved and substituted into the trained GRNN. Thus, the intrusive data were classified. The paper selected a GRNN that was separately optimized using a genetic algorithm (GA), particle swarm optimization (PSO), and fuzzy C-mean clustering (FCM) to enable a comparison of these approaches. As shown in the results, the AIAE-GRNN achieves a higher classification accuracy than PSO-GRNN, but the running time of AIAE-GRNN is long, which was proved first. FCM and GA-GRNN were eliminated because of their deficiencies in terms of accuracy and convergence. To improve the running speed, the paper adopted principal component analysis (PCA) to reduce the dimensions of the intrusive data. With the reduction in dimensionality, the PCA-AIAE-GRNN decreases in accuracy less and has better convergence than the PCA-PSO-GRNN, and the running speed of the PCA-AIAE-GRNN was relatively improved. The experimental results show that the AIAE-GRNN has a higher robustness and accuracy than the other algorithms considered and can thus be used to classify the intrusive data.

## Introduction

Network security has become increasingly significant with the development of the Internet. Because of the diversity and complexity of intrusion methods, it is difficult to effectively detect the traditional firewall technique. Network intrusion detection, such as neural networks, appeared at a historic moment [1–3]. The essence of intrusion detection is a classifying problem in which the data of several keys of the Internet or systems are analyzed, the data are classified as normal or abnormal parts using the clustering abilities of neural networks, and a decision is made as to whether the security policy has been broken. Early methods of intrusion detection include techniques based on probabilistic and statistical analyses [4–6] and expert systems in intrusion detection [7, 8]. For example, Forrest S. et al. proposed an intrusion detection method based on comparing the statistical characteristics of the sequences that were called by the systems between the normal behaviors and abnormal behaviors [5]; Warrender C. et al. built a hidden Markov model for the running status of computer systems [6]; Ilgun K. et al. proposed STAT/NSTAT (state transition analysis tool/netstat) based on expert experience to detect the intrusion data [8]; Lindqvist U. et al. introduced an intrusion detection method called P-BEST (production-based expert system toolset) based on a set of rules [7]. Compared with these traditional methods, the applications of neural networks in intrusion detection have many merits. Although methods based on probabilistic and statistical analyses are more mature, they are insensitive to the orders of productions of the intrusions, and the threshold value cannot be accurately ensured. In contrast, because the intrusion activities have more characteristics, the number of their dimensionalities is typically more than 30, and the performance of the traditional statistical analyses will be worse in processing high-dimension data, which causes a high error rate in classifying the intrusion data [9, 10]. Although the approaches based on expert systems have high accuracy, the cost of establishing and maintaining the rule bases is high [4]. Thus, the current main studies on building the structure of expert systems are machine learning and data mining. The neural network is an important part of artificial intelligence and data mining, where back propagation (BP) and radial basis function (RBF) neural networks are the most widely applied. BP neural networks are a type of multi-layer perceptron artificial neural network that can handle a complex non-linear problem, which consists of an input layer, hidden layer, and output layer. However, it has some intrinsic defects; namely, the performance is highly related to the topology structure (the number of hidden layer nodal points) and initial parameters (including the initial connection weights and thresholds of connection nodes), and it easily falls into a local optimal solution [11]. The RBF neural network is similar to the BP neural network. Both networks approach the error by adjusting the weights of neurons [12]. However, the RBF neural network differs from the BP network in some respects. The number of nodes in the hidden layer of the BP network cannot be easily identified, whereas the RBF network can adjust the number of nodes in its hidden layer according to the specific problems; thus, its adaptability is better. Nevertheless, the RBF and BP networks unfortunately have the same defects: low convergence rate and easily falling into a local optimal solution [13]. The RBF network is typically applied in nonlinear time series prediction instead of data classification [13–16]. In contrast, the general regression neural network (GRNN) performs well in nonlinear mapping and has an excellent approximate capability; thus, it overcomes the defects of the traditional BP and RBF networks, such as easily falling into the local optimal solutions and low convergence speed, which makes it suitable for solving the problems of nonlinear functions [13]. Because of these characteristics, the GRNN is often used for data regression and classification and has achieved good results [17–20].

The traditional intelligent algorithms of data regression and classification, such as the traditional GRNN [21], fuzzy C-means clustering (FCM) [22], the GA-GRNN algorithm [23], which combines the genetic algorithm (GA) and GRNN, and the PSO-GRNN algorithm [19], which combines particle swarm optimization (PSO) and the GRNN, have some deficiencies. For example, artificial factors will negatively affect the accuracy of the traditional GRNN because the smooth factors that affect its accuracy are difficult to confirm using experiences or trials. FCM becomes easily trapped in local optimum solutions, and it is difficult to classify [22] because there are many dimensions of the network intrusion characters and the different data categories are similar. In contrast, the performance of FCM and the classification result are affected by the weighting exponent of the FCM, and the traditional FCM cannot guarantee that it obtains the optimal values of the weighting exponent [24]. Thus, the classification may not have the highest accuracy. The GA-GRNN and PSO-GRNN have the same deficiencies as FCM because the data dimensions are high and they have bad local search abilities, which result in premature convergence [25, 26]. The artificial immune algorithm (AIA) uses the diverse generation and maintenance mechanism of immune systems to maintain diverse optimal solutions, and thus, it overcomes the premature convergence of the general optimization processes, particularly the multi-peak functions, and allows global optimum solutions to be obtained. These characters give the AIA higher robustness in solving complex problems [27, 28]. The AIA has been applied in the data-mining field [29]. However, the AIA also has some deficiencies, such as a slow running speed and convergence rate [30]. To overcome these deficiencies, the artificial immune algorithm with elitist strategies (AIAE) was introduced [31]. The elitist archive and elitist crossover were added to the traditional AIA, which improved the adaptivity and accuracy of the algorithm [32, 33].

This study was devoted to exploring a new combinatorial neural network to rapidly determine the intrusion types. Both GRNN and AIAE perform better than their related algorithms. Their combination may produce better results and improve the efficiency of intrusion detection. In addition, the combination of the AIA and neural networks is an important part of algorithm studies [34]. Currently, research on the combination of these two algorithms remains sparse. This study combined AIAE and GRNN and used the excellent global convergence ability of the AIAE to optimize the smooth factors of the GRNN. The optimal smooth factor consists of the optimal structure of the GRNN. Thus, the network intrusion data can be accurately classified, and a better intrusion detection method is obtained.

## Materials and Methods

### General regression neural network (GRNN)

GRNN is a radial basis function neural network that is composed of an input layer, pattern layer, summation layer and output layer. Its theoretical basis is the nonlinear regression analysis. The essence of the regression analysis, which is composed of the independent variable *Y* and dependent variable *x*, is to calculate *y*, which has the maximum probability values. Assuming that *f*(*x*,*y*) is the joint probability density function of the random variables *x* and *y*, the observed value of *x* is *X*. Thus, the regression of *y* relative to *X* is

$$\hat{Y}=E\left(y|x\right)=\frac{\int_{-\infty }^{+\infty }yf(x,y)dy}{\int_{-\infty }^{+\infty }f(x,y)dy}$$ (1)

where $\hat{Y}$ is the forecast output of *Y* when the input is *X*.

The density function $\hat{f}(X,y)$ can be estimated based on the sample data set ${\left\{x_{i},y_{i}\right\}}_{i=1}^{n}$ using Parzen non-parametric estimation:

$$\hat{f}(X,y)=\frac{1}{n{\left(2\pi \right)}^{\frac{p+1}{2}}\sigma^{p+1}}\sum_{i=1}^{n}\exp[-\frac{{\left(X-X_{i}\right)}^{T}(X-X_{i})}{2\sigma^{2}}]\exp[-\frac{{\left(X-Y_{i}\right)}^{2}}{2\sigma^{2}}]$$ (2)

where *X*_*i*_ and *Y*_*i*_ are the observed values of the random variables *x* and *y*; *n* is the sample size; *p* is the dimension of *x*; and *σ* is the width coefficient of the Gaussian function, which is called the smooth factor.

*f*(*x*,*y*) is substituted by $\hat{f}(X,y)$ in Eq. 1, and the orders of the integrals and additions are swapped:

$$\hat{Y}\left(X\right)=\frac{\sum_{i=1}^{n}\exp[-\frac{{\left(X-X_{i}\right)}^{T}(X-X_{i})}{2\sigma^{2}}]\int_{-\infty }^{+\infty }y\exp[-\frac{{\left(Y-Y_{i}\right)}^{2}}{2\sigma^{2}}]dy}{\sum_{i=1}^{n}\exp[-\frac{{\left(X-X_{i}\right)}^{T}(X-X_{i})}{2\sigma^{2}}]\int_{-\infty }^{+\infty }\exp[-\frac{{\left(Y-Y_{i}\right)}^{2}}{2\sigma^{2}}]dy}$$ (3)

Because$\int_{-\infty }^{+\infty }ze^{-z^{2}}dz=0$, the output $\hat{Y}(X)$ of the network after the two integrals are calculated is

$$\hat{Y}\left(X\right)=\frac{\sum_{i=1}^{n}Y_{i}\exp[-\frac{{\left(X-X_{i}\right)}^{T}(X-X_{i})}{2\sigma^{2}}]}{\sum_{i=1}^{n}\exp[-\frac{{\left(X-X_{i}\right)}^{T}(X-X_{i})}{2\sigma^{2}}]}$$ (4)

$\hat{Y}(X)$is the weighted average of all sample observed values *Y*_*i*_. Each weight factor of *Y*_*i*_ is the index of the squared Euclidean distance between *X*_*i*_ and *X*. When *σ* is notably large, $\hat{Y}(X)$is similar to the average of the dependent variables of all samples. In contrast, when *σ* approaches 0, $\hat{Y}(X)$is highly similar to the training samples. If the predicted points are contained in the training sample set, the predictions are highly similar to the corresponding dependent variables in the samples. However, if the points are not contained in the samples, the predictive effect is notably bad, which demonstrates the poor generalization capability of the network. Thus, the optimization of *σ* directly determines the predictive accuracy of the GRNN [13, 21].

### Immune algorithm with elitist strategies

The AIA and GA have similar structures: they consist of the operations of selection, crossover and mutation. Unlike the GA, the quality of individuals (antibodies) is evaluated using the affinity (fitness) and concentration in the AIA, which reflects the diversities of the real immune systems. However, the only evaluation index of the GA is the fitness [35]. Thus, the AIA evaluates the individuals more comprehensively than the GA. Hajiaghaei-Keshteli M defined the parameters of the AIA, which included the antibodies’ similarity, concentration, expected breed rate, selection probability and diversity indices [35].

This study used the elitist-archive and elitist-crossover strategies to prevent the loss of the current optimal individuals in the next generation of the population.

- Definition 1: In the elitist-archive strategy, the individuals with the maximum fitness, which are called the elitists, must not perform the selection and mutation operations when the experiment begins. The elitists will survive with the selective probability of 100% in the next generation, and they will not be damaged by the selection, crossover and mutation operations. To maintain the scale of the population, the individuals with the minimum fitness will be eliminated if the elitists are added into the next generation [32].
- Definition 2: In the elitist-crossover strategy, the elitists should be crossed with the chosen individuals in the population according to a pre-set elitist-crossover probability in addition to the traditional crossover; thus, the genetic structure of the population can be improved. The theory supporting this strategy is provided in Tan GZ et al. [33]. The number of excellent patterns can be increased using the elitist crossover. However, the diversity of the population will decrease if the algorithm only adopts the elitist crossover. Thus, the traditional crossover should be used in combination with the elitist crossover so that it maintains the population diversity and improves the total population quality.

### Principal component analysis

Principal component analysis (PCA) is a type of technology that can analyze and simplify data sets. The dimensions of the data sets can be decreased by reserving the low-order principal components and neglecting the high-order ones; simultaneously, the characteristics with the largest contribution for the variances in the data sets are reserved. Such low-order components can reserve the most important aspects of data. However, because the PCA is extremely sensitive to extrema and missing values, these factors may produce missing or incorrect results [36, 37]. Thus, it will negatively affect the study of neural networks and the classification accuracy.

### Methods

**Fig 1:**
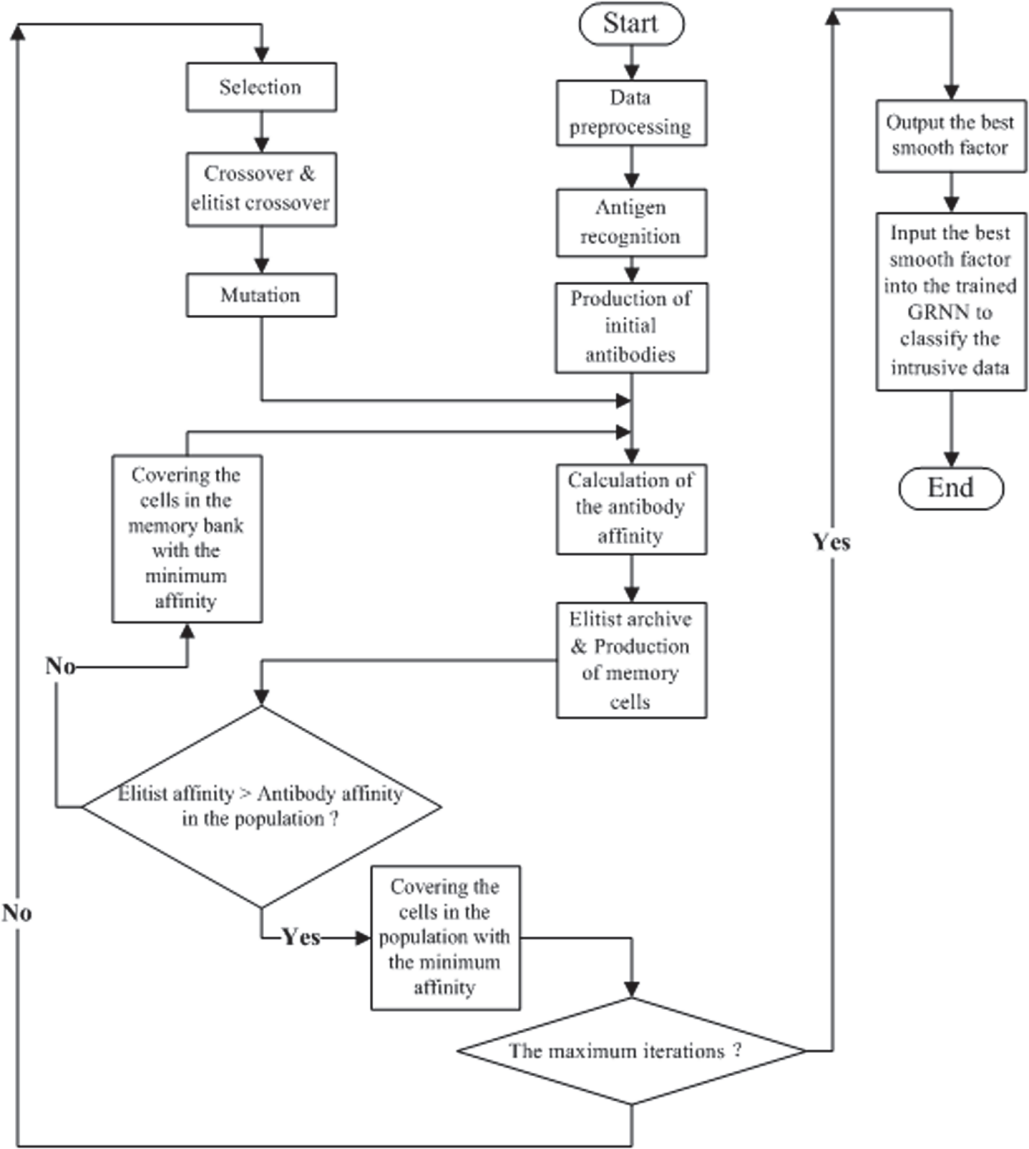
The algorithm flow chart of AIAE-GRNN.

This paper introduces an AIAE-GRNN algorithm to improve the detection accuracy. The mean square errors (MSEs) of the network model created by the GRNN were taken as the affinity functions. The smooth factors were optimized by iterating the AIAE until the algorithm satisfied the terminal condition. The smooth factor that was solved at this time is the optimal one. Then, the GRNN with the optimal smooth factor was used to classify the intrusion data. The algorithm flow chart is shown in Fig. 1. The concrete steps are:

- Step 1: Data preprocessing. To eliminate the magnitude differences among the data of each dimension and improve the classification accuracy, the intrusion data could be normalized into the figures in [−1, 1] [38]. Because there were 38 indices in the input section of the intrusion data, the PCA could be used in the dimensionality reduction. However, the accuracy may decline because the PCA only extracts characteristics with the largest contributions to the variances in the data and neglects some secondary characteristics. This problem will be discussed later.
- Step 2: Antigen recognition. The affinity functions, which were the antigens in the immune system, were set as the MSEs of the output values and actual values in each generation of the GRNN. The initial parameters of the antigens, such as the population size *N*, crossover probability *P*_*c*_, elitist-crossover probability *P*_*ck*_ and mutation probability *P*_*v*_, were set. The running parameters of the algorithm, such as the maximum iterations and capacity of the memory bank, were also set.
- Step 3: Production of the initial antibodies. *N* smooth factors were randomly produced as the initial solutions.
- Step 4: Calculation of the antibody affinities. The intrusive data were plugged into the GRNN after normalization; then, the output values of the MSE in this generation were produced.
- Step 5: Elitist archive. The current individuals with the largest affinities were preserved in the memory bank as memory cells in a certain proportion. Each antibody affinity was calculated, and if all current antibodies had lower affinities than elitists, the antibodies with the lowest affinities would be replaced by the elitists. Then, the algorithm continued to Step 6. In contrast, if some antibodies had higher affinities than the elitists, these antibodies would replace the elitists with the lowest affinities in the population; then, the algorithm would repeat Step 4.
- Step 6: Judgment of the loop's termination condition. If the algorithm achieved the maximum iterations, it would be terminated and output the current smooth factor. Then, the smooth factor would be an input of the trained GRNN to classify the intrusive data. Otherwise, the algorithm would continue to Step 7.
- Step 7: Calculation of the antibody concentration and expected breed rate. Each antibody concentration and expected breed rate would be calculated according to the computing methods of Hajiaghaei-Keshteli M [35].
- Step 8: Selection. The algorithm would perform the selection operation. The probability of the individuals under selection was the expected breed rate, which had been calculated.
- Step 9: Crossover and elitist crossover. The population would be crossed with probability *P*_*c*_; then, the elitist-crossover operation would be performed on the individuals in the population using the methods of the previous study [33], and the probability *P*_*ck*_ of the operation was notably low.
- Step 10: Mutation. The mutative positions, which were randomly selected, would be mutated with probability *P*_*k*_; then, the affinities of the variant individuals would return to Step 4 to be calculated again.

## Simulation and Testing

### Data

The experimental environment was based on Microsoft Windows 7. The hardware environment was Intel Core i5-3230M, 2.60 GHz and RAM 4 GB, and the simulation software was MATLAB 2012b (MathWorks, located in Natick, Massachusetts, U.S.A.). The experimental data were divided into the training data (S1 Data) and the testing data (S2 Data). All the data were obtained from the network intrusion data set KDD CUP 1999 [39], which included normal data (Normal) and four categories of abnormal data of a denial-of-service attack (DoS), probing attack (PROBE), remote-to-login attack (R2L) and user-to-root attack (U2R). The input of this data set has 38 indices, and its output has one index, which is the abnormal category. The training and testing samples are shown in S1 Table.

### Contrast models and evaluation indices

In addition to the AIAE-GRNN, the study selected two common GRNN combinational algorithms, GA-GRNN and PSO-GRNN, as the controlled trials, and FCM was the control sample. In fact, the convergence of FCM is directly related to the selection of the weighting exponent. The value of the weighting exponent in the traditional FCM must be chosen by experience; thus, the human factors will seriously affect the performance of the FCM [24]. This factor has a less significant effect on convergence compared to other algorithms. Similarly, the traditional GRNN was not selected, because it is identical to the FCM, where the smooth factor may not be the optimal one. Therefore, the FCM was only selected as a referential control, and the convergence of FCM was not discussed. In addition, some papers [40, 41] have proven that the convergence of the traditional FCM is not sufficient, and the traditional FCM typically has to be combined with other algorithms, such as a GA. Thus, FCM is not used by itself, and it is not selected to compare the convergence with other algorithms. The evaluation indices were the detection rate (DR), false positive rate (FPR), maximum iterations and running time [2, 3]. The algorithms were tested after being classified according to the abnormal categories. Before the formal test, a number of preliminary tests were necessary, and reasonable experimental parameters were set according to previously published work [42–44]. Thus, a better experimental result was obtained, and the running time was not excessively large. The maximum number of iterations was 100; the population size *N* of each algorithm was 30; the crossover probability *P*_*c*_ and mutation probability *P*_*v*_ of the AIAE and GA were 0.5; the two acceleration factors of PSO, i.e., *c*_1_ and *c*_2_, were 2; the AIAE evaluation diversity was 0.95; the capacity of the memory bank was 10; and the elitist-crossover probability *P*_*ck*_ was 0.05. The definitions of DR and FPR are as follows [45]:

- Definition 3: The detection rate (DR) is the probability that the detection systems can correctly give an alarm when the monitoring systems are attacked.
- Definition 4: The false positive rate (FPR) is the probability that the normal instructions are mistakenly considered intrusion instructions, and an alarm is given.

### Results and further discussion

**Fig 2:**
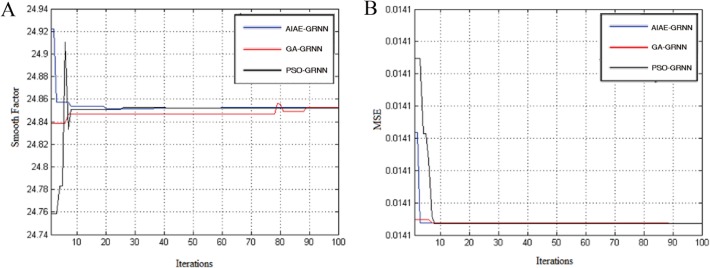
The output performances of GA-GRNN, PSO-GRNN and AIAE-GRNN. (A) The relationship between the optimized GRNN smooth factors and the iterations of GA-GRNN, PSO-GRNN and AIAE-GRNN. (B) The relationship between MSE and the iterations of GA-GRNN, PSO-GRNN and AIAE-GRNN.

Table 1 shows the DR and FPR of the algorithms, and Table 2 shows the relationship between the convergence iterations and the running time. Fig. 2A shows the relationship between the optimized GRNN smooth factors and the iterations, and Fig. 2B shows the relationship between the MSE and number of iterations.

**Table 1:** DR & FPR of each intrusion category by the different algorithms.

Categories	DoS		R2L		Probe		U2R	
Methods	DR	FPR	DR	FPR	DR	FPR	DR	FPR
GA-GRNN	99.43	0.36	98	0.15	99.06	0.56	98.32	0.22
PSO-GRNN	99.54	0.23	97.59	0.11	99.2	0.49	98.75	0.21
AIAE-GRNN	99.88	0.05	97.98	0.06	99.44	0.36	98.89	0.16
FCM	90.53	2.65	56.48	0.63	82.67	1.96	76.88	0.23

The result showed that the DR and FPR of PSO-GRNN and AIAE-GRNN were higher than GA-GRNN and FCM. And the DR and FPR of AIAE-GRNN were higher slightly than PSO-GRNN.

**Table 2:** The evaluation indexes for the different algorithms.

Methods	GA-GRNN	PSO-GRNN	AIAE-GRNN	FCM
Fitness	0.0141	0.0141	0.0141	—
Sooth factors	24.8551	24.8522	24.8542	—
Convergence generations	89	8	8	—
Running time	19min38s	19min20s	29min24s	8s

The result showed that the GA-GRNN had the premature convergence problem. In contrast, the PSO-GRNN and AIAE-GRNN overcame this problem. The running time of PSO-GRNN was shorter than AIAE-GRNN.

Table 1 shows that the AIAE-GRNN has the highest DR, but its running time is the longest because of the high algorithm complexity of the AIAE [30, 46–48]. Although the PSO-GRNN has a slightly lower DR than the AIAE-GRNN, it has the same convergence rate and a faster running speed than the AIAE-GRNN. Their ratio of running time is 0.6576. As shown in Fig. 2A, the GA-GRNN appears to have a premature convergence. During the 78^th^ iteration, the values of the GRNN smooth factors continue to change by a large margin, which shows that it has a worse local search ability than the AIAE-GRNN and PSO-GRNN and a lower classification accuracy than the AIAE-GRNN. Although the traditional FCM has a short running time, its classification accuracy is not comparable with other intelligence algorithms, which results in a difficult classification (Table 1). Thus, both the AIAE-GRNN and PSO-GRNN have merits and defects, and further experiments were necessary to test the performance of these two algorithms. However, the GA-GRNN and FCM were eliminated.

PCA was used to test the two remaining algorithms (AIAE-GRNN and PSO-GRNN). PCA can reduce the dimensions of the training samples and training time [49], but the classification accuracy may be affected. Thus, it was necessary to test this approach using experiments. PCA was performed for the input data using SPSS 19. Fourteen types of input evaluation indices had the largest eigenvalues, with a cumulative contribution of 86.42% determined using the SPSS 19 analysis. When the cumulative contribution is above 85%, the largest eigenvalues can represent the principal characteristics of the entire data set [50]. Thus, these 14 input indices were chosen as the training and testing samples.

**Fig 3:**
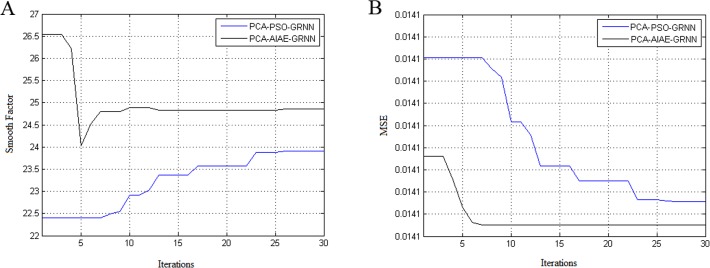
The output performances of PCA-AIAE-GRNN and PCA-PSO-GRNN. (A) The relationship between the optimized GRNN smooth factors and the iterations of PCA-AIAE-GRNN and PCA-PSO-GRNN. (B) The relationship between MSE and the iterations of PCA-AIAE-GRNN and PCA-PSO-GRNN.

The value of the maximum iteration was 30, and other parameters were identical to those in the previous paragraphs. The DR and FPR of the two networks are shown in Table 3, and the convergence iterations and running time are shown in Table 4. The relationship between the optimized GRNN smooth factors and the iterations is shown in Fig. 3A, and the relationship between MSE and the iterations is shown in Fig. 3B.

**Table 3:** DR & FPR of each intrusion category by PCA-PSO-GRNN and PCA-AIAE-GRNN.

Categories	DoS		R2L		Probe		U2R	
Methods	DR	FPR	DR	FPR	DR	FPR	DR	FPR
PCA-PSO-GRNN	97.74	0.35	95.88	0.04	97.5	0.45	98.81	0.15
PCA-AIAE-GRNN	99.41	0.01	98.4	0	99.01	0.22	98.45	0.03

By reducing dimensions in PCA, compared with Table 1, the DR and FPR of PSO-GRNN and AIAE-GRNN declined to a certain extent, but the DR and FPR of AIAE-GRNN was still higher than PSO-GRNN.

**Table 4:** The evaluation indexes for PCA-PSO-GRNN and PCA-AIAE-GRNN.

Methods	PSO-GRNN	AIAE-GRNN
Fitness	0.0141	0.0141
Sooth factors	23.4547	24.8546
Convergence generations	23	7
Running time	3min58s	6min3s

By reducing dimensions in PCA, compared with Table 2, the convergence and relative running time were improved. This result showed that the robustness of AIAE-GRNN was better than PSO-GRNN.

From Table 2, compared with the network without PCA, the DRs of the 4 intrusion categories that were optimized using PCA-AIAE-GRNN decrease by only 0–0.5%, but all FPRs are below those of the previous AIAE-GRNN. However, the DR of the 4 intrusion categories that were optimized using PCA-PSO-GRNN decreases by 1.7–2.1%; in particular, the R2L has the largest decrease range of 2.12%, and only the DR of U2R increases slightly. Their running time ratio is 0.6556, which is lower than the ratio without PCA. This phenomenon indicates that the PCA-AIAE-GRNN has a faster running speed than the previous networks. Figs. 3A & B show that the PCA-PSO-GRNN has worse convergence than the PCA-AIAE-GRNN and appears to have a premature convergence, which indicates a poor local search ability. The results of further experiments show that the AIAE-GRNN has better robustness than the PSO-GRNN, and the accuracy is not strongly affected by the application of PCA.

## Conclusion

The study introduced a method of network intrusion detection using the AIAE-GRNN. The AIAE-GRNN has a higher classification accuracy but longer running time than two types of GRNN combinational algorithms and FCM. To decrease the running time and prove the robustness of the network, this study used PCA to reduce the dimensions of the intrusive data, which effectively reduced the training time of the network. In the contrast test, which contained PSO-GRNN that was similarly processed with PCA, the classification accuracy of AIAE-GRNN with the shortened running time is barely affected, and the AIAE-GRNN has better convergence than the PSO-GRNN. This result indicates that the AIAE-GRNN is not notably sensitive for the training samples and shows a good robustness. The PCA-AIAE-GRNN balances the classification accuracy and running efficiency, and can therefore be used in intrusion detection or other fields. Although the AIAE-GRNN has many merits, the AIAE has a lower running speed than PSO and other intelligence algorithms. Optimizing the time complexity of the AIAE-GRNN will be critical to improving the network performance and must be studied further.

## Supporting Information

S1 Table The training and testing samples for the networks. (DOC)

S1 Dataset The training data. (XLS)

S2 Dataset The testing data. (XLS)
