# Supplementary material for: Network Intrusion Detection Based on a General Regression Neural Network Optimized by an Improved Artificial Immune Algorithm
Source: PLoS One. 2015 Mar 25;10(3):e0120976. doi: 10.1371/journal.pone.0120976 (PMC4373783; doi:10.1371/journal.pone.0120976)
Supplement: S1 Table — (DOC) [file pone.0120976.s001.doc]

**S1 Table The training and testing samples for the networks**

| Categories | Attack types | Training Number | Testing Number |
| --- | --- | --- | --- |
| DoS | smurf | 487 | 631 |
|  | neptune | 218 | 290 |
|  | back | 238 | 341 |
|  | teardrop | 190 | 228 |
|  | pod | 121 | 15 |
|  | land | 16 | 83 |
| R2L | warezclient | 108 | 362 |
|  | warezmaster | 3 | 19 |
|  | spy | 2 | 3 |
|  | guess_passwd | 34 | 17 |
|  | ftp_write | 4 | 43 |
|  | imap | 10 | 7 |
|  | multihop | 4 | 2 |
|  | phf | 7 | 2 |
| Probe | ipsweep | 117 | 461 |
|  | satan | 165 | 338 |
|  | portsweep | 99 | 41 |
|  | nmap | 28 | 30 |
| U2R | buffer_overﬂow | 122 | 219 |
|  | rootkit | 34 | 105 |
|  | loadmodule | 22 | 44 |
|  | perl | 17 | 26 |
| Normal |  | 368 | 1283 |
| Total | | 2414 | 4590 |

The categories of the intrusion include DoS, R2L, Probe and U2R. The normal state is labeled as Normal.
